# Case Report: Esophageal cancer under comprehensive treatment strategy—application and clinical outcome analysis of combined immunotherapy, targeted therapy, and low-dose radiotherapy

**DOI:** 10.3389/fonc.2025.1510371

**Published:** 2025-03-28

**Authors:** Yanling Yuan, Xingxi Pan, Lihua Tong, Wanming He, Wen Yang

**Affiliations:** Department of Oncology, The Sixth Affiliated Hospital, School of Medicine, South China University of Technology, Foshan, Guangdong, China

**Keywords:** esophageal carcinoma, immune checkpoint inhibitor, programmed cell death protein 1, vascular endothelial growth factor receptor, low dose rate radiotherapy

## Abstract

Current evidence for the combined use of immunotherapy, low-dose chemoradiotherapy, and epidermal growth factor receptor-targeted therapy for the treatment of advanced esophageal squamous cell carcinoma is lacking. We report the case of a 73-year-old woman with squamous cell carcinoma of upper middle thoracic esophagus. After undergoing concurrent chemoradiotherapy combined with immunotherapy and anti-angiogenic targeted therapy, the patient achieved a progression-free survival of 17 months. To date, the patient has achieved 35 months of overall survival, which continues to extend, with a good quality of life. Immunotherapy combined with low-dose concurrent chemoradiotherapy is a promising option for elderly patients with advanced esophageal cancer who are intolerant to standard treatments. The addition of an epidermal growth factor receptor monoclonal antibody as a radiosensitizer improves therapeutic efficacy. The combination of sintilimab and anlotinib has the potential to treat recurrent and metastatic esophageal cancer. Tailoring treatment strategies for specific patient groups is essential in personalized medicine.

## 1 Introduction

Esophageal cancer is the sixth leading cause of cancer-related deaths (1). Over half of the new esophageal cancer cases occur in China (2), with esophageal squamous cell carcinoma (ESCC) being the predominant pathological type (3). Available treatment options include surgery, chemotherapy, radiotherapy, molecular-targeted therapy, and their combinations. However, the prognosis remains poor, with a very low overall 5-year survival rate, especially among older people (3). Although achieving effective treatment remains challenging, recent advances in immunotherapy and targeted therapies have demonstrated promise for improving outcomes (4–6).

Concurrent chemoradiotherapy (CRT) remains the cornerstone treatment for locally advanced esophageal cancer, providing superior local tumor control and survival benefits compared to radiotherapy alone (7–9). The CROSS trial established paclitaxel plus carboplatin as a preferred definitive CRT regimen, demonstrating improved overall survival (OS) and disease-specific survival in both preoperative and unresectable settings (10, 11). Additionally, the RTOG 85-01 trial confirmed the benefits of CRT with fluorouracil plus cisplatin, reporting a median survival advantage (14 vs. 9 months) and significantly improved 5-year OS (27% vs. 0%) over radiotherapy alone, with sustained survival benefits at 8 and 10 years. CRT also enhances local tumor control, reducing local failure rates (47% vs. 65%) compared to radiotherapy alone. These findings underscore CRT’s pivotal role in improving long-term outcomes in esophageal cancer (7, 8).However, CRT-related toxicities, particularly radiation-induced esophagitis and myelosuppression, often lead to treatment discontinuation or suboptimal dosing, limiting its efficacy in elderly or frail patients (12–14). In recent years, the integration of immunotherapy and targeted therapy has emerged as a strategy to enhance tumor response and mitigate resistance mechanisms (6, 15). Preclinical and clinical studies suggest that EGFR-targeted agents, such as nimotuzumab—an anti-EGFR monoclonal antibody—may enhance radiosensitivity (16, 17). Despite these advancements, the optimal strategy for combining CRT with immunotherapy and targeted agents remains undefined, necessitating further clinical investigation into novel treatment paradigms.

This case report describes the use of sintilimab, low-dose-rate chemoradiotherapy, and nimotuzumab for the curative treatment of an older patient with advanced esophageal squamous cell carcinoma, followed by maintenance therapy with immunotherapy and anti-angiogenesis treatment. In this case, immunotherapy played a central role throughout treatment. The use of low-dose radiotherapy helped reduce radiotherapy-related side effects and improve patient tolerance. Preclinical studies suggest that LDRT enhances immune responses by promoting tumor antigen release, increasing T cell infiltration, and modifying the tumor microenvironment, potentially improving tumor control when combined with immunotherapy (18). Recent clinical studies have further supported these findings, demonstrating that radiotherapy plus PD-1 inhibitors significantly improve progression-free and overall survival in advanced solid tumors (19). These data reinforce the potential of combining LDRT with immunotherapy to optimize treatment efficacy in esophageal cancer. Additionally, the concurrent use of albumin-bound paclitaxel and nimotuzumab as radiosensitizing agents during weekly radiotherapy sessions contributed significantly to improving chemotherapy sensitivity. The patient achieved progression-free survival for 17 months and survived for over 35 months without reaching the endpoint. These findings suggest the potential of this combination to improve efficacy while maintaining tolerability, particularly in older patients.

## 2 Case description

### 2.1 Primary concerns

A 73-year-old woman was admitted to our hospital on October 15, 2021, due to the diagnosis of esophageal cancer for more than 1 year and dysphagia with chest and back pain for more than 1 month.

### 2.2 History of past illness

The patient was in good health and denied having any pre-existing diseases, such as hypertension and diabetes.

### 2.3 Personal and family history

The family background did not include a history of similar tumors, nor were there any instances of cardiovascular or cerebrovascular diseases.

### 2.4 History of present illness

In September 2020, the patient was diagnosed with poorly differentiated esophageal squamous cell carcinoma. Chest computed tomography (CT) revealed esophageal wall thickening and lymph node metastasis. The patient received anlotinib (12 mg, days 1-14) as anti-angiogenesis therapy. In August 2021, follow-up showed tumor progression, and immunosuppressive therapy was recommended but declined. By September 2021, her swallowing difficulty worsened, accompanied by chest and back pain, leading to hospital admission for further evaluation and treatment.

### 2.5 Physical examination

The Karnofsky Performance Scale and Numeric Rating Scale scores were 70 and 3, respectively. Enlarged lymph nodes were palpable in the bilateral supraclavicular regions and were more prominent on the right side, where the lymph nodes were fused, with the largest measuring approximately 3 × 4 cm, having a hard texture, and exhibiting poor mobility. No enlarged lymph nodes were palpable. Bilateral lung breath sounds were normal and no wheezing was detected. The heart rhythm was regular and no pathological murmurs were heard in the valve auscultation areas.

### 2.6 Laboratory examinations

The complete blood count, liver and kidney function, coagulation function, and tumor marker levels were normal.

### 2.7 Imaging examinations

**Figure 1:**
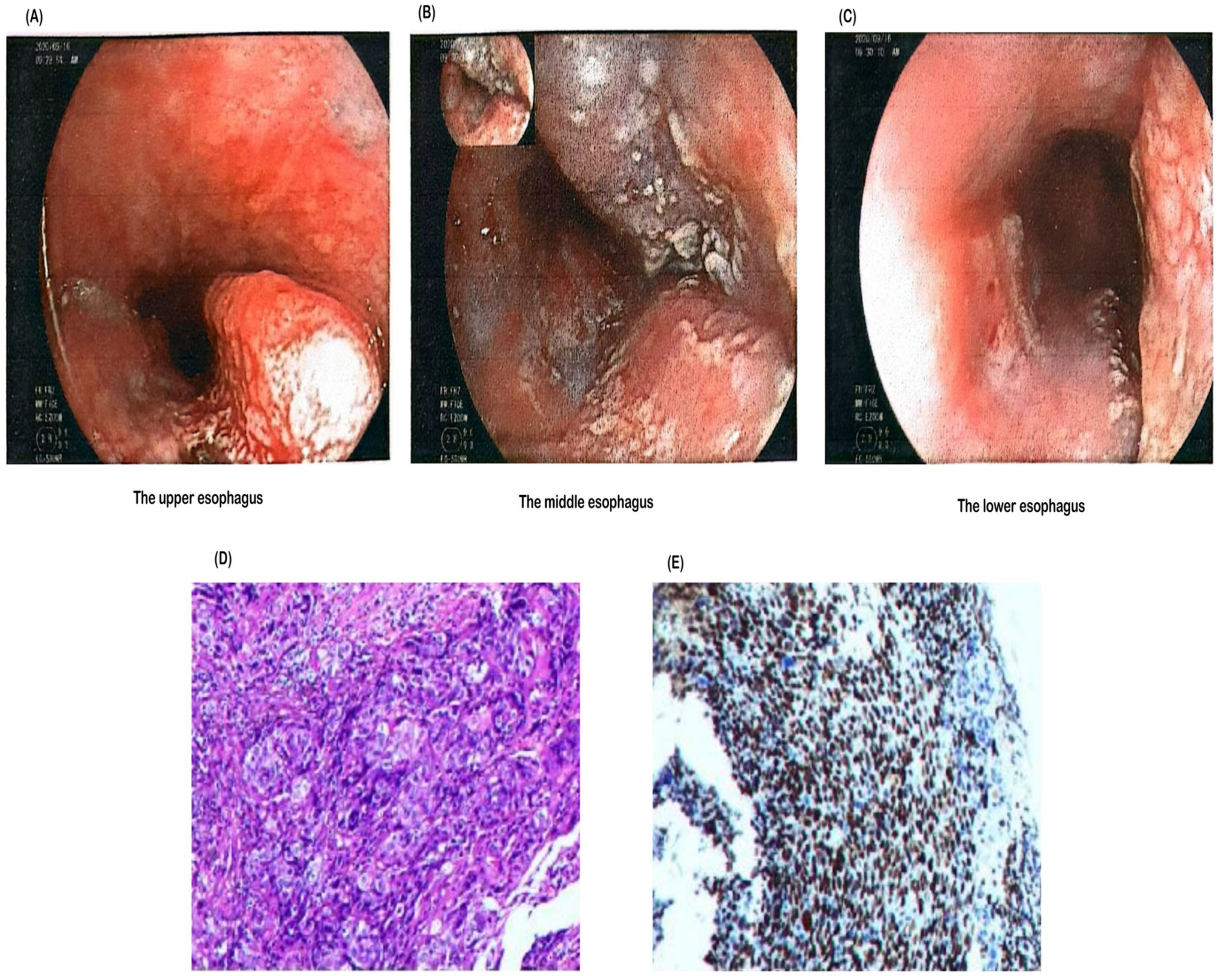
Endoscopic and histopathological findings of the esophageal lesion. **(A)** Endoscopic image showing a lesion in the upper esophagus. **(B)** A tumor was observed in the middle esophagus, approximately 17–24 cm from the incisor. The lesion circumferentially involved the lumen, appeared brittle, and bled easily upon contact. **(C)** Another tumor was found in the lower esophagus, approximately 30–35 cm from the incisor. The lesion occupied about half of the lumen, was friable, and bled easily upon contact. **(D)** Hematoxylin and eosin (H&E) staining of the biopsy specimen demonstrating malignant squamous epithelial cells with irregular nuclei and high mitotic activity, indicative of poorly differentiated squamous cell carcinoma. **(E)** Immunohistochemical staining of the biopsy specimen showing strong nuclear expression of p40, confirming the diagnosis of squamous cell carcinoma.

**Figure 2:**
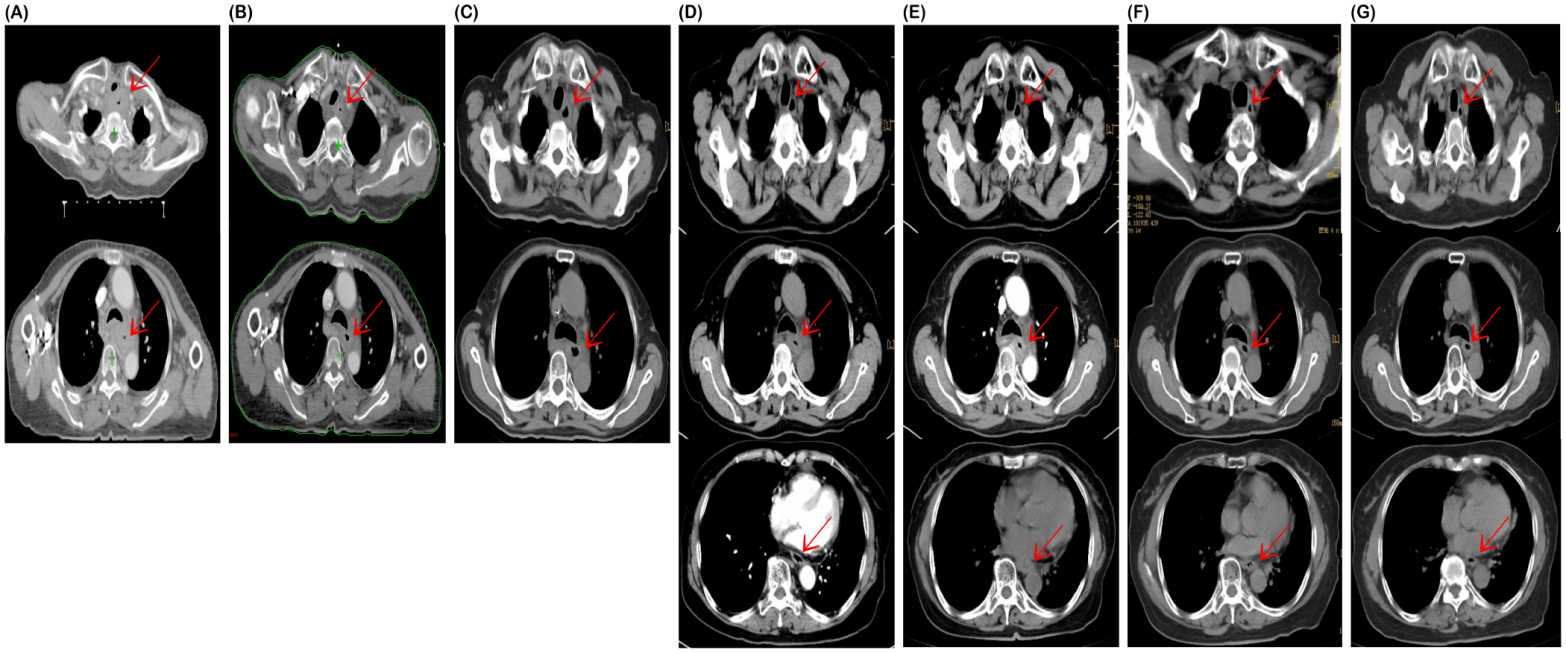
Computed tomography (CT) at the start of treatment on October 20, 2021 **(A)**, offline ART-based CT on November 17, 2021 **(B)**, and post-radiotherapy assessment CT on January 29, 2022 **(C)**, showed progressive tumor shrinkage. Computed tomography (CT) on June 16, 2022 **(D)**, after maintenance therapy showed stable disease (SD); on March 24, 2023 **(E)**, thoracic lesions remained stable, but esophageal thickening indicated tumor progression. Following treatment with sintilimab and anlotinib, chest CT scans on January 23, 2024 **(F)**, and April 16, 2024 **(G)**, indicated continued partial response.

1. Gastroscopy performed at Nanhai District Public Health Hospital of Foshan City (**Figures 1A-C**) revealed an esophageal mass. Further immunohistochemistry was recommended.
2. Pathology performed at Guangzhou Kangdu Clinical Testing Center (**Figures 1D, E**): (Esophagus 17-21 cm from incisors) Based on microscopic and immunohistochemical results, the lesion was consistent with poorly differentiated esophageal squamous cell carcinoma. Immunohistochemical results were as follows: P40 (+++), Ki-67 (50%+), P63 (++), and CK5/6 (++).
3. Contrast-enhanced CT of the chest and whole abdomen (**Figure 2A**) revealed irregular thickening of the esophageal wall at the cervical and upper-middle thoracic segments, with multiple enlarged lymph nodes in the surrounding area, bilateral supraclavicular fossa, and mediastinum, suggesting esophageal cancer with multiple lymph node metastases. Poor demarcation of the posterior tracheal margin was noted.

### 2.8 Final diagnosis

Clinical staging was defined according to the 8th edition of the American Joint Committee on Cancer Tumour, Node, Metastasis staging system, combined with clinical, imaging, and pathological diagnosis results, which indicated that the tumor invaded the trachea, metastasized to the periesophageal, supraclavicular, and mediastinal lymph nodes, and showed no distant metastasis. The patient was clinically diagnosed with ESCC of the cervical and upper middle thoracic esophagus (cT4N3M0, stage IVA, G3).

### 2.9 Treatment, outcome, and follow-up

**Figure 3:**
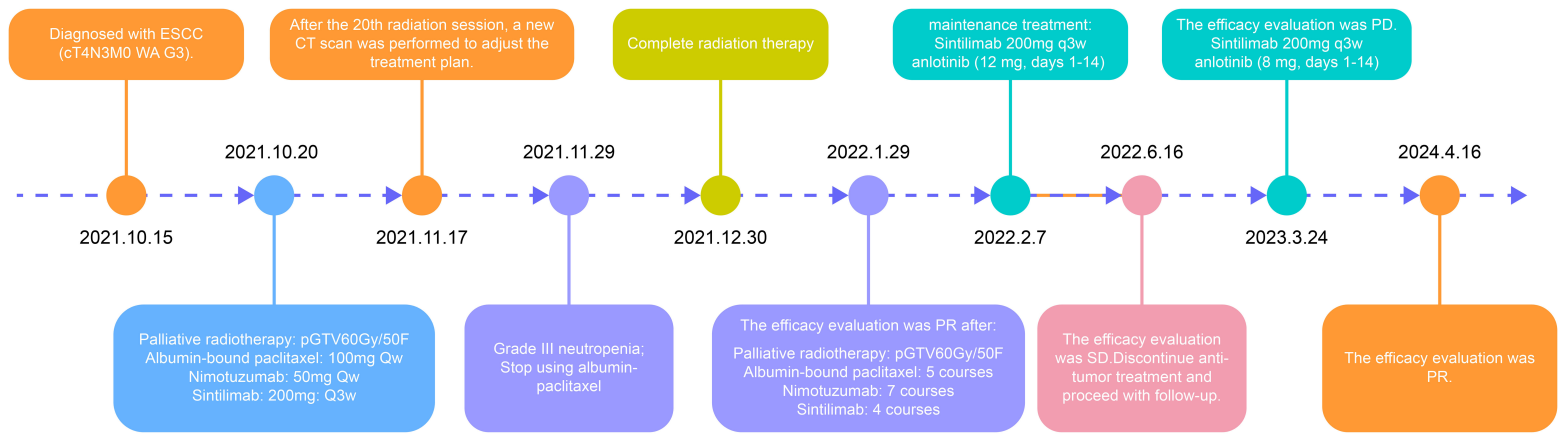
The flowchart of the treatment process of the patient.

In accordance with the treatment guidelines at the time, the experience of the physician, and the preferences of the patient and family, concurrent chemoradiotherapy combined with nimotuzumab for radiosensitization and immunotherapy with sintilimab was administered. Given the advanced age of the patient, the comprehensive treatment approach, and financial considerations, albumin-bound paclitaxel and nimotuzumab were administered at a reduced dose. The specific treatment process is illustrated in **Figure 3**.

The patient was admitted with difficulty in swallowing. The adaptive radiation therapy-based CT imaging after 20 radiotherapy sessions (**Figure 2B**) and and post-radiotherapy assessment CT imaging (**Figure 2C**) showed significant tumor shrinkage, and the difficulty in swallowing improved markedly. In June 2022, a follow-up chest CT showed stable disease (**Figure 2D**), and the patient completed maintenance therapy. A follow-up CT in March 2023 revealed continued tumor regression within the radiation field but recurrence in the lower esophagus (**Figure 2E**). Recurrence occurred more than six months later. Treatment with a combination of sintilimab and anlotinib (8 mg) was resumed. Chest CT scans performed on January 23 (**Figure 2F**) and April 16 (**Figure 2G**), 2024, showed a continued partial response.

**Figure 4:**
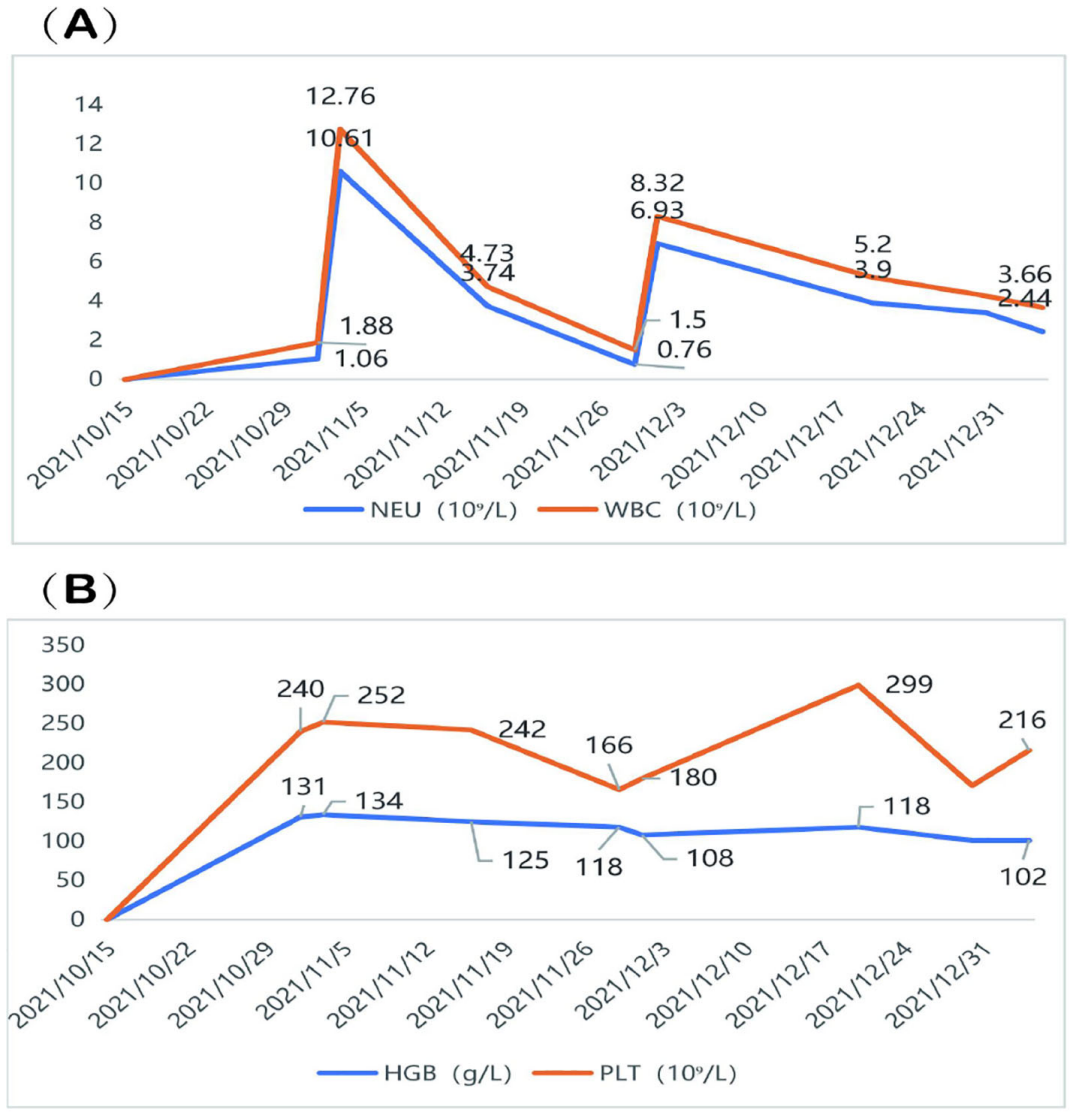
Hematologic changes during treatment. **(A)** The patient developed Grade III neutropenia, with the lowest WBC count of 1.50 ×10^9^/L and NEU count of 0.76 ×10^9^/L recorded on November 29, 2021. **(B)** A mild decline in HGB and PLT was observed, but no severe thrombocytopenia or anemia occurred. WBC, White blood cell; NEU, neutrophil; HGB, Hemoglobin; PLT, platelet.

During concurrent chemoradiotherapy and targeted therapy, the patient experienced hematologic toxicity, primarily manifesting as leukopenia and neutropenia. The lowest WBC count (1.50 ×10^9^/L) and neutrophil count (0.76 ×10^9^/L) were recorded on November 29, 2021, consistent with Grade III neutropenia. In response, albumin-bound paclitaxel was discontinued, and granulocyte colony-stimulating factor (G-CSF) therapy was initiated, leading to hematologic recovery. Mild decreases in hemoglobin and platelet counts were observed, but no further episodes of severe myelosuppression occurred during the subsequent course of radiotherapy combined with targeted therapy (**Figure 4**). Grade II radiation esophagitis was managed with oral oxycodone extended-release tablets (20 mg every 12 h), which provided relief, and the patient was able to consume a liquid diet. No other significant side effects were observed and no dosage adjustments were made during treatment.

## 3 Patient perspective

On September 26, 2024, during a follow-up with the family of the patient, they reported that the patient was very satisfied with the treatment outcomes. The current condition was stable, and the patient could eat normally on their own, although the swallowing speed was slightly reduced.

## 4 Discussion

The treatment of advanced ESCC remains a significant clinical challenge owing to the poor prognosis and limited efficacy of conventional therapies. In recent years, the advent of immunotherapy, particularly immune checkpoint inhibitors targeting programmed cell death protein-1/programmed death-ligand 1 pathway, has provided a new therapeutic avenue for patients with advanced ESCC (4, 5, 20).

Definitive concurrent chemoradiotherapy has been suggested as the primary treatment for patients with unresectable disease or for those who are medically unfit for surgery. In the setting of Definitive concurrent chemoradiotherapy, the Radiation Therapy Oncology Group 85-01 trial recommended 50 Gy in 25 fractions as the standard radiation dose (8, 21). According to the 2019 Chinese Society of Clinical Oncologyguidelines, radiotherapy or chemoradiotherapy is still advised for older patients or those with poor cardiopulmonary function or multiple comorbidities who are not candidates for surgery. Radiotherapy is an effective option for alleviating clinical symptoms such as bleeding, pain, and dysphagia in advanced ESCC and for improving the quality of life, nutritional status, and survival outcomes. One study confirmed that in older patients with esophageal carcinoma who are not candidates for surgery, concurrent chemoradiotherapy with a maximum total radiation dose of 63 Gy (5 ×1.8 Gy/week) results in significantly better treatment outcomes than radiotherapy alone (22).

In the present study, definitive chemoradiotherapy was initially considered. However, the extensive involvement of the tumor, including airway invasion, along with a history of anlotinib treatment and significant dysphagia, posed challenges. The patient refused a gastrostomy for nutritional support, and conventional fractionated chemoradiotherapy carries a high risk of esophagotracheal fistula formation. Additionally, concern existed that the patient might not tolerate the side effects of the treatment, potentially leading to discontinuation.

According to previous studies, low-dose radiotherapy combined with modified chemotherapy using a continuous low-dose regimen can improve treatment compliance and promote fistula healing in patients with esophageal cancer complicated by esophagotracheal fistulas (23, 24). Low-dose radiotherapy of 60 Gy (5 ×1.2 Gy/week) in 50 fractions was administered in the present study, reducing the per-fraction dose and extending the treatment duration to prevent the tumor from shrinking too rapidly while allowing for normal tissue repair. This approach helped to minimize the risk of acute radiation-induced mucositis and perforation. Our goal was to ensure that a sufficiently high cumulative dose would still offer a chance for tumor control. Older patients have poor tolerance to standard dual-drug chemotherapy; therefore, for concurrent chemotherapy, weekly albumin-bound paclitaxel at 100 mg was used owing to its relatively low toxicity profile (25).

Preclinical studies have revealed that Nimotuzumab, a humanized monoclonal antibody that targets epidermal growth factor receptor, enhances radiosensitivity by promoting radiation-induced apoptosis (26). In the intent-to-treat population, the endoscopic complete response rates were 47.2% with nimotuzumab and 33.3% without nimotuzumab (P = 0.17), whereas the combined endoscopic complete response and pathologic complete response rates were significantly higher with nimotuzumab at 62.3% compared to 37.0% without it (P = 0.02). Nimotuzumab combined with chemoradiotherapy can achieve better complete response rates, extend survival, and does not increase adverse effects (16, 27). Recent findings suggest that the combination of nimotuzumab with radiotherapy is well tolerated and produces favorable outcomes in older patients with unresectable esophageal cancer (17). To further enhance the radiosensitization effect of the treatment and considering the financial capacity of the patient, we added nimotuzumab 50 mg/week was added as a targeted therapy in this study.

Recent breakthroughs in immunotherapy have presented new avenues for the treatment of advanced ESCC (4, 5, 20). Studies such as KEYNOTE-181 and ATTRACTION-03 have demonstrated significant survival benefits of programmed cell death protein -1 inhibitors, including pembrolizumab and nivolumab, establishing immunotherapy as a viable option for these patients (4, 5). The ORIENT-15 trial further demonstrated that sintilimab combined with chemotherapy offered superior survival outcomes compared with chemotherapy alone, regardless A of programmed death-ligand 1 expression (20). In accordance with the treatment guidelines, second-line sintilimab immunotherapy combined with chemoradiotherapy is recommended. Therefore, in the present study, sintilimab immunotherapy was added in combination with chemoradiotherapy and targeted therapy.

Antiangiogenic therapies are becoming integral to the treatment of esophageal cancer. Anlotinib, a selective Vascular Endothelial Growth Factor receptor 2 inhibitor, has demonstrated strong anti-angiogenic and antitumor effects in preclinical models (28).Based on the ALTER1102 study (29), the 2019 Chinese Society of Clinical Oncology Eophageal Cancer Guidelines recommend anlotinib monotherapy as second-line and beyond in advanced esophageal squamous cell carcinoma. When combined with immunotherapy, anlotinib can reshape the tumor microenvironment and enhance immune cell infiltration and treatment efficacy, showing promise in esophageal cancer and other malignancies (6, 15).At the end of radiotherapy, partial response was achieved according to the Response Evaluation Criteria in Solid tumors criteria 1.1 (30). As a result, a combination of sintilimab and anlotinib maintenance immunotherapy was initiated in January 2022 and continued until June 2022 owing to financial constraints. The tumor recurred in March 2023 and treatment with sintilimab and anlotinib remained effective, with continued disease regression observed in April 2024. A follow-up on September 26, 2024, revealed that the patient was well, with no pain and normal eating ability.

This case report highlights the successful use of low-dose radiotherapy combined with immunotherapy in a patient with stage IVA esophageal cancer, achieving a progression-free survival of 17 months with ongoing overall survival. This treatment minimizes radiotherapy-related side effects, improves tolerability, and enables high-quality survival. Although the financial limitations of the patient required reduced doses of nimotuzumab, this comprehensive strategy proved to be effective. However, additional limitations should be considered. The interaction between LDRT, immunotherapy, and targeted therapy may vary among patients, potentially influencing immune responses. Additionally, while myelosuppression was not severe, mild radiation-induced esophagitis and fatigue were observed, requiring supportive care. Further research is needed to refine the balance between maximizing therapeutic efficacy and minimizing adverse effects.

Due to the financial constraints and poor compliance of the patient, testing for programmed death-ligand 1 and epidermal growth factor receptor expression was incomplete. During the later follow-up period, the patient refused hospitalization and completed only outpatient CT scans. After May 2024, the patient returned to their hometown and was not followed up at our hospital, leaving incomplete imaging data. Current status updates are available only via phone, and further studies are required to confirm the long-term benefits and broader applicability of this approach.

## Data Availability

The original contributions presented in the study are included in the article/supplementary material. Further inquiries can be directed to the corresponding author.
